# Private Rooms in Fever Hospitals

**Published:** 1910-11-12

**Authors:** 


### II.—PRIVATE ROOMS IN FEVER HOSPITALS.

The Chairman : The next subject in the question-box is "Should private rooms be provided in fever hospitals for patients who are prepared to pay for them? Can they be attended by their own medical man?" I ask Dr. Ebenezer Duncan, who has always something worthy of hearing, to speak upon this question.

Dr. Ebenezer Duncan : Mr. Chairman, ladies and gentlemen, this question which has been put down on the paper is one about which there can be considerable difference of opinion, but I shall endeavour to be as brief as possible. The provision of private rooms for paying patients in our fever hospitals has been advocated ever since these institutions were rendered necessary by the clauses of the Public Health Acts which apply to the prevention and mitigation of infectious and contagious maladies. I have myself raised the question on several occasions many years ago. The Public Health Act does not make it compulsory for those who have proper means of isolation at home to go to a fever hospital, and it would therefore be incorrect to say that all persons, whether they can or cannot pay for private rooms in a fever hospital, are compelled to go there. Nevertheless, even a millionaire living in an hotel, or a person of moderate means in a lodging-house, when seized by an infectious malady is under compulsion to go to the rate-paid hospital and submit to all its inconveniences and dangers. Modern fever hospitals provided by the public rates and free to all are looked upon with justifiable pride as beneficent examples of the advance of preventive medicine, and I would be the last to deny their utility and convenience. They are a great boon to the poor. They prevent a great deal of inconvenience and suffering. They save a great many lives, and I have no doubt they have an influence in preventing the spread of some epidemic diseases; but why should those who are willing to pay for special accommodation go upon the rates? Some of them object, and even if the only objection to send their children to a rate-paid hospital is that either from principle or from prejudice they do not desire to take their child from a refined home to be placed in a cot next to a child from the slums, with its objectionable habits and language, is that not a reasonable objection? But that is by no means the greatest reason for desiring isolation in private apartments at any reasonable cost. The patients may be subjected to a much more serious contamination. They may get their disease aggravated by septic infection from an adjoining bed. They may go with one disease and get another. Cross infection and mixed infection from septic cases are not uncommon. The reports of even our most modern fever hospitals built on the pavilion plan prove this, and until provision is made for isolation of every case it must continue to prevail more or less. I do not think it fair that a mild case of scarlet fever should be treated in a bed next to a severe case with a septic sore throat. I do not think a mild case of erysipelas should be treated in the same ward with a virulent case, violently delirious and noisy. In order to free rate-paid hospitals from cross infection and septic infection, and from the inconvenience caused by noisy and offensive patients, large wards must give place to separate cubicles such as in the South-Western Fever Hospital, Stockwell, or in Walthamstow Hospital, London. This will lead to greater expenditure both in building, in equipment, and in nursing. The expense per patient will be increased and the accommodation will be diminished. I am glad to say that in Glasgow isolation blocks are being built with small rooms for septic and doubtful cases, and plans are being considered for the trial of the cubicle system. I hope that in considering these plans effect may be given to the demand for private rooms for pay patients. But how is the additional expense per patient and the diminished accommodation to be compensated? I believe there is plenty of room in the existing hospitals in Glasgow for all legitimate cases, and plenty of room for the provision of private rooms for paying patients. Already attempts have been made to convert the existing large wards of isolation fever hospitals into cubicles with the object of preventing mixed infection and providing accommodation for the isolation of doubtful cases and of septic cases. At the South-Western Fever Hospital, Stockwell, two large wards were converted into cubicles about three years ago. Each ward, which originally contained eighteen beds, was divided by glass partitions, framed in iron and reaching to a height of 7 feet above the floor level, into sixteen cubicles, 120 square feet being enclosed within each cubicle. Every cubicle is furnished with a fixed basin, over which is fixed a tepid spray worked by pedal action. A portable enamelled bath is supplied, and a gas-heated copper steriliser for sterilising every article which has been used for the patient's treatment. In the scullery attached to the ward is a second steriliser of larger size for the disinfection of plates, mugs, knives, forks, and spoons after they have been removed from the cubicles. Separate overalls are hung in each of the cubicles, which are worn by the nursing and medical staff attending that particular patient, and a sterilised towel hung in proximity to the spray basin, which provides for the ablution of the hands of the attendants. A second and more complete arrangement of a similar kind has been carried out at the Walthamstow hospital for infectious diseases. The last point is that private paying patients should have, if they desire it, their own medical adviser to attend them in fever hospitals. That is better for the patient, and it need not interfere with the administration, as proper rules as to hours and special nursing, etc., would avoid all friction, whereas I think that mere admission as consultants would not be so good.

